# The use of oral fluids to monitor key pathogens in porcine respiratory disease complex

**DOI:** 10.1186/s40813-017-0055-4

**Published:** 2017-04-05

**Authors:** Juan Hernandez-Garcia, Nardy Robben, Damien Magnée, Thomas Eley, Ian Dennis, Sara M. Kayes, Jill R. Thomson, Alexander W. Tucker

**Affiliations:** 1 Department of Veterinary Medicine, University of Cambridge, Madingley Road, CB30ES Cambridge, England, UK; 2 Thermo Fisher Scientific, Waltham, MA USA; 3 Royal Veterinary College, University of London, London, England, UK; 4 BQP Ltd., Stradbroke, England, UK; 5 SAC Consulting Veterinary, Scotland’s Rural College (SRUC), Penicuik, Midlothian Scotland, UK

**Keywords:** Oral fluids, PCV2, *Mycoplasma hyopneumoniae*, *SIV*, *PRRSV*

## Abstract

**Background:**

The usefulness of oral fluid (OF) sampling for surveillance of infections in pig populations is already accepted but its value as a tool to support investigations of porcine respiratory disease complex (PRDC) has been less well studied. This study set out to describe detection patterns of porcine reproductive and respiratory syndrome virus (PRRSV), porcine circovirus type 2 (PCV2), swine influenza virus type A (SIV) and *Mycoplasma hyopneumoniae* (*M. hyo*) among farms showing differing severity of PRDC.

The study included six wean-to-finish pig batches from farms with historical occurrence of respiratory disease. OF samples were collected from six pens every two weeks from the 5^th^ to the 21^st^ week of age and tested by real time PCR for presence of PRRSV, SIV and *M. hyo* and by quantitative real time PCR for PCV2. Data was evaluated alongside clinical and post-mortem observations, mortality rate, slaughter pathology, histopathology, and immunohistochemistry testing data for PCV2 antigen where available.

**Results:**

PRRSV and *M. hyo* were detectable in OF but with inconsistency between pens at the same sampling time and within pens over sequential sampling times. Detection of SIV in clinical and subclinical cases showed good consistency between pens at the same sampling time point with detection possible for periods of 2–4 weeks. Quantitative testing of OF for PCV2 indicated different patterns and levels of detection between farms unaffected or affected by porcine circovirus diseases (PCVD). There was good correlation of PCR results for multiple samples collected from the same pen but no associations were found between prevalence of positive test results and pen location in the building or sex of pigs.

**Conclusions:**

Detection patterns for PRRSV, SIV and *M. hyo* supported the effectiveness of OF testing as an additional tool for diagnostic investigation of PRDC but emphasised the importance of sampling from multiple pens and on multiple occasions. Preliminary evidence supported the measurement of PCV2 load in pooled OF as a tool for prediction of clinical or subclinical PCVD at farm level.

## Background

Respiratory disease results in major losses in the pig industry through reduced performance, increased mortality and antimicrobial use [1, 2] with negative impacts on animal welfare and public health. Multiple pathogens contribute to a polymicrobial infection known as Porcine Respiratory Disease Complex (PRDC) [3]. These pathogens can be classified as primary agents, which overcome and weaken the host defence mechanisms, or opportunistic secondary pathogens that take advantage of impaired defences resulting in aggravated disease often requiring longer periods of antimicrobial treatment. Key primary agents of PRDC include porcine reproductive and respiratory syndrome virus (PRRSV), *Mycoplasma hyopneumoniae* (*M. hyo*), swine influenza virus (SIV) and porcine circovirus type 2 (PCV2) [4]. Combined infection with PRDC-associated pathogens can produce synergistic effects via mechanisms that include producing immune depression, alteration of macrophage function and cytokine response, and hampering of the mucociliary clearance in the respiratory tract which enables bacterial colonization [3]. Such interactions have been described in the literature between PCV2 and other agents [4, 5], also for PRRSV [6–9], SIV [10], *M. hyo* [11, 12], and further respiratory pathogens [3].

Diagnostic investigation of PRDC at population and individual level is complicated by this polymicrobial nature of the problem but also by the dynamic progression over time, with different pathogens being dominant or detectable at different stages of the disease process [3]. Laboratory techniques have become a core element of the diagnostic process for PRDC, supplementing clinical examination, and pathological findings; A wide range of different diagnostic samples and techniques may be employed in a single case investigation, including for example antibody detection in serum, antigen detection or culture from respiratory tract fluids and tissue samples [13]. Recently, oral fluid (OF) has been used as an additional sample platform for antibody and nucleic acid detection of key PRDC pathogens [14].

The commercial use of OF-based diagnostics has grown considerably in recent years because of practical, economic and welfare advantages of collecting OF rather than serum. It is important to note that OF may also contain material from the respiratory system, environmental and faecal contamination, material from the nasal cavity, antibodies and crevicular fluid, further widening the scope for detection of infectious agents through this sample platform but also resulting in some laboratory challenges. Problems related to polymerase chain reaction (PCR) inhibitors in OF and sample degradation have been major limitations for this technique [15, 16]. However, knowledge surrounding laboratory diagnostic techniques based on OF has increased markedly since 2010 [17] including refined collection methods [18, 19] and processing [20] or preservation methods [15, 21] to optimise sample quality. In addition, optimised PCR conditions have been developed for specific respiratory pathogens including PRRSV [22, 23], SIV [24, 25] and PCV2 [26, 27]. Most focus has been on viral rather than bacterial pathogen detection in OF but the knowledge base is increasing for *Actinobacillus pleuropneumoniae*, *Haemophilus parasuis* [28] and *M. hyo* [29–31]. In addition, the OF platform relies on a pooled sample at pen-level from animals that may or may not interact individually with the sampling rope and may themselves be shedding pathogen, or have antibody titres, at differing levels thereby raising potential constraints related to sensitivity of the test. The availability of data on the wide scale use of OF testing to investigate PRDC, and the implications for these concerns, remains scarce and there are only a few studies investigating multiple PRDC pathogens on the same samples [14, 32, 33].

This study set out to describe the detection of key respiratory pathogens in OF samples collected over time from commercial pig populations undergoing respiratory disease. This was achieved by a longitudinal study of PRRSV, PCV2, SIV and *M. hyo* detection in OF alongside clinical and pathological information. Although the usefulness of OF sampling for surveillance of pig populations has already been described [14], the present study set out to describe differences in detection patterns between farms more or less severely affected by PRDC, thereby providing initial insights into the usefulness of OF sampling in diagnostic investigations, in addition to its proven value as a surveillance tool.

## Methods

### Study design

A longitudinal survey was carried out over an eight-month period (January to August 2015) using six batches of pigs. Study batches were selected from three different breeding sources of commercial crossbred pigs from a single production pyramid. These sources were classified as low (A), medium (B) and high risk for suffering respiratory problems (C) based on the severity and incidence of historical respiratory problems, clinical observations, post mortem examinations, laboratory results and slaughter pathology evaluations during the wean-to-finish phase.

Two batches per source were selected for study making a total of six batches. Each batch was assigned at weaning (24–30 days of age) to a wean-to-finish farm: Pigs from source A (low severity of respiratory problems) were allocated in farm A1 and A2, pigs from source B (medium severity of respiratory problems) went to farm B1 and B2, and finally, pigs from source C (high severity or respiratory problems) were sent to farms C1 and C2 (Table 1). At arrival, pigs were segregated by gender and randomly allocated in pens (30 to 120 pigs/pen). Farms were composed by several barns, but just one barn per farm was considered for testing purposes. Each barn had pens arranged in a row with solid floors comprising a straw bedded area and a dunging area allowing for removal of faeces using a tractor-based scrape-through passage system. Consequently, each barn was divided into three sections: clean (pen 1 and 2 in the furthest point form the passage exit), central (pen 3 and 4 at the mid-way point along the passage) and dirty (pen 5 and 6 near the passage exit). One male pig pen and one female pig pen were selected in each section making a total of six pens per batch that were repeatedly sampled in OF at 2-week intervals across nine time points (in weeks 5, 7, 9, 11, 13, 15, 17, 19 and 21 of age) starting in the week after arrival and finishing in the 21^st^ week of age a few weeks prior to slaughter. Every batch was vaccinated one to seven days after weaning against PCV2 and *M. hyo*. Pigs in C1 and B2 were also vaccinated against PRRSV at the 5^th^ week of age.

**Table 1:** Pig batches included in the study with reported respiratory problems in previous batches

Source (expected severity of respiratory problems)	A (low)	B (medium)	C (high)
Expected problems based on historical records.	Source negative for PRRSV and *M. hyo*. Occasional problems with SIV.	Source positive for PRRSV but negative for *M. hyo*. Previous batches presented SIV and *Streptococcus suis* disease.	Source positive for PRRSV and *M. hyo*. Previous batches presented respiratory and *Streptococcus suis* disease.

### Samples and data collection

#### Batch clinical observations and casualties

Clinical observation data was collected during each sampling visit with categorisation of clinical signs and external lesions (respiratory, enteric, neurological, tail bitten, wasting, musculoskeletal, found dead/unknown) considering severity and incidence. Where possible, veterinary post-mortem examination was done to support the categorisation and to permit sample collection for extended investigations. Where justified by clinical or post mortem findings, PCVD diagnosis was done based on published criteria [34] on lung and inguinal lymph node tissue fixed in 10% formalin and processed for histopathological evaluation and immunohistochemistry by Cap protein specific PCV2 monoclonal antibody (INGENASA, Madrid, Spain) [35]. Wean to finish mortality data was collected for each batch.

#### Batch slaughter pathology data

A minimum of 10% of the pigs of each batch were examined at slaughter (without consideration of whether pigs originated from pens previously surveyed, or not, by OF); lungs were evaluated following the British Pig Executive Pig Health Scheme (BPHS) scoring system for average severity of enzootic pneumonia (EP)-like lesions and prevalence of pleurisy lesions based in the Goodwin score system [36, 37].

#### OF and serum samples

OF samples were collected with unbleached cotton ropes using a ratio of one rope for each 25 pigs with a 30 min exposure. Ropes were 1.5 cm thick, and 20 cm long for young pigs and 40 cm long for pigs older than 17 week-old. Ropes were hung in central parts of the pen when possible so pigs had 360° access, distributed in different points to maximize interactions. The height of the bottom part of the rope was adjusted to fit match with the average height of the pigs’ shoulder joint.

Ropes were collected into individual containers and shipped under chilled conditions for next day delivery to the diagnostic laboratory. Blood samples were collected from 12 pigs in each of the six batches at 15 weeks of age as part of the farms’ routine health monitoring program; these pigs were randomly selected among the six OF tested pens.

### Laboratory analysis of OF and serum

Serum samples and OF samples collected from each rope were individually analysed and no pools were done. OF samples were stored at 4 °C after arrival and nucleic acids were extracted on the day of receipt. Residual OF was stored at −80 °C. Total nucleic acids were extracted using the MagMAX™ Express-96 Particle Processor (MME-96; Thermo Fisher Scientific) and the MagMAX™ -96 Viral RNA Isolation Kit (cat. no. AM1836; Thermo Fisher Scientific) following the manufacturer’s OF Sample Extraction Protocol in the MagMAX™ Pathogen RNA/DNA Kit manual. Extracted nucleic acids were stored at −80 °C prior to testing by PCR.

Nucleic acid samples were tested for four porcine pathogens. PCV2, SIV and PRRSV were tested using commercial real time PCR kits (LSI VetMAX™ Porcine Circovirus Type 2 – Quantification kit, VetMAX™ Gold SIV Detection Kit, and LSI VetMAX™ PRRSV EU/NA, Thermo Fisher Scientific). The presence of *M. hyo* was determined using VetMAX™ *M. hyopneumoniae* reagents paired with VetMAX™ -plus quantitative PCR (qPCR) Master Mix (Thermo Fisher Scientific). The SIV, PRRSV and *M. hyo* assays were presence/absence tests only while the PCV2 PCR was supplied with a quantified control that allowed the quantification of viral genome copies in positive samples (genome copies/mL OF). SIV positive samples were subtyped using H1H3 Duplex and N1N2 Duplex primer/probe mixes (still in development in 2015; Thermo Fisher Scientific) in conjunction with Path-ID™ Multiplex One-Step RT-PCR Kit (Thermo Fisher Scientific). All PCRs were prepared as per the kit inserts and were run on a 7500 Real Time PCR System (Applied Biosystems). Data was analysed using the 7500 Software.

Extracted serum was tested for antibodies against PRRSV strains of genotype 1 and 2 (IDEXX PRRS X3 5/STRIP, IDEXX), real-time PCR for PRRSV (LSI VetMAX™ PRRSV EU/NA, Thermo Fisher Scientific) and by qPCR for PCV2 LSI VetMAX™ Porcine Circovirus Type 2 – Quantification kit, Thermo Fisher Scientific).

Values of cycle threshold (Ct) below 37 were considered as positives in the case of PRRSV, Ct results between 37 and 40 were re-analysed according to the manufacturer recommendations, and considered as positive if Ct values in the second analysis were lower than 40. For SIV and *M. hyo*, Ct values below 37 were considered positive and values between 37 and 40 were reported as weak positives, according to the manufacturer recommendations, though only positives with Ct < 37 are considered in most of the downstream analysis as detailed above. In the case of PCV2, values for log10 genome copies/mL were calculated from the raw Ct values using the internal standard curve, which enables accurate quantification in a range between 1 × 10^4^ and 1 × 10^8^ genome copies/mL. Limits of detection for samples extracted with MagMAX™ Pathogen RNA/DNA and analysed with LSI VetMAX™ PCV2 were set in 1 × 10^3.48^ genome copies/mL for OF, and 1 × 10^3.6^ genome copies/mL for blood or serum. Results under the limit of detection were considered inconclusive, and viral load results out of the quantification range were expected to be inaccurate.

### Data analysis

Ct values and viral load results of each rope collected were individually analysed and interpreted considering clinical signs, post-mortem lesions, histopathological findings and slaughterhouse evaluations. Agreement among results from different ropes in a pen, and different pens in a barn were assessed to estimate the robustness of this sampling method in this scenario. Pens and barns were considered positive for a pathogen where at least one of the OF samples resulted positive for PRRS, with a Ct value <37 for SIV and *M. hyo*, or presented a PCV2 viral load over the limit of detection (1 × 10^3.48^ genome copies/mL).

Correlations between Ct values for multiple OF samples collected from the same pen were evaluated using Pearson’s correlation coefficient. In the case more than two ropes in a pen presented a Ct value <40, highest and lowest Ct values were selected for the analysis. Statistics were performed by using R version 3.3.1 [38].

## Results

### Clinical signs, causes of death and post-mortem investigations

Observations for clinical signs among the batches were grouped into three stages of production: nursery (aged 5–8 weeks), grower (age 9–15 weeks) and finisher (age 16 weeks to slaughter at around 23 weeks) (Table 2). Three out the six batches presented signs of respiratory disease commencing after weaning in A1, B2 and C1, and these continued for all or most of the growing and finishing period. In the case of B1 and C2, respiratory problems started later at the grower stage. Signs of enteric disease were observed soon after weaning in 3 of the batches namely B1, B2 and C1 but only in B2 did these signs continue into the grower phase. Neurological signs associated with suspected meningitis were observed in four of the batches with A1 and C1 experiencing problems during the nursery period but B1 and B2 experiencing problems commencing in the grower stage. Wasting was noted only in B2, throughout the grower and finisher stages, while more general uneven growth was found in B2 but also in the finisher stage of C1. No clinical signs were observed in A2.

**Table 2:** Clinical observations recorded during 2-weekly sampling visits and slaughter lung evaluation

	Weeks 5 to 8	Weeks 9 to 15	Week 16 to finish	Pigs evaluated at slaughter/Number of pigs in the batch at weaning	Average EP-like lesion score at slaughter	Prevalence (%) of pleurisy lesions at slaughter
A1	Respiratory (+) Neurological (+)	Respiratory (++)	Respiratory (++)	250/786	1.2	12
A2	No signs observed	No signs observed	No signs observed	248/1005	1.4	1
B1	Enteric (+)	Respiratory (+) Neurological (++)	Respiratory (+) Tail biting. (+)	202/1204	1.4	6
B2	Enteric (+++) Respiratory (++)	Neurological (+), Enteric (+) Respiratory (+++), Wasting (++) Uneven growth (+++)	Respiratory (++) Wasting (+) Uneven growth (+++)	126/1063	7.8	1
C1	Respiratory (+) Neurological (+) Enteric (+)	Respiratory (++) Musculoskeletal (+)	Uneven growth (++) Musculoskeletal (+) Respiratory (+)	163/709	4.7	1
C2	No signs observed	Respiratory (+)	Respiratory (+++)	157/1105	1.9	2

“+” indicates an estimation of a low proportion (<15%) of affected pigs, “++” a moderate proportion (15–35%), and “+++” a high proportion (>35%) of affected pigs. EP-Like lesions based on the BPHS system based on the Goodwin scale [36]

**Fig. 1:**
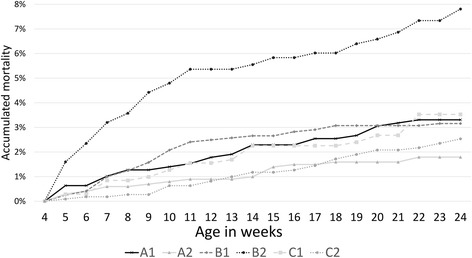
Cumulative mortality per batch. Cumulative mortality, according to pig age, comprising pigs that died or were euthanized among the study batches from weaning until slaughter

Casualty pigs, comprised of pigs that died or were euthanized, were categorised into broad suspected causal groups (Table 3). Data were dependent on stockperson categorisation and as such should be interpreted with caution, in particular the category of ‘found dead’, but a total of 48 of 220 casualties underwent post mortem examination by a veterinarian and these categorisations were confirmed. Respiratory causes were the most frequent, or jointly most frequent, recorded reason for casualty in all batches except A2, and wean to finish mortality, defined as casualty pigs that died or were euthanized, ranged from 1.8% (A2) to 7.7% (B2) (Table 3, Fig. 1).

**Table 3:** Categorisation of the suspected cause of death for dead and euthanized pigs

Cause recorded	Number of pigs per batch (and percentage of the total in the batch)					
	A1 *N* = 786	A2 *N* = 1005	B1 *N* = 1204	B2 *N* = 1063	C1 *N* = 709	C2 *N* = 1105
Respiratory	22 (2.7%)	0	16 (1.3%)	27 (2.5%)	6 (0.8%)	7 (0.6%)
Enteric	0	0	0	1 (0.1%)	0	0
Neurological	0	1 (0.1%)	7 (0.6%)	21 (2%)	3 (0.4%)	1 (0.1%)
Tail bitten	0	5 (0.5%)	0	0	0	0
Wasting	0	1 (0.1%)	0	16 (1.5%)	0	4 (0.3%)
Musculoskeletal	0	1 (0.1%)	2 (0.2%)	5 (0.5%)	1 (0.1%)	7 (0.6%)
Found dead/unknown	4 (0.5%)	10 (1%)	13 (1.1%)	13 (1.2%)	15 (2.1%)	9 (0.8%)
Total of casualties	26 (3.3%)	18 (1.8%)	38 (3.2%)	83 (7.8%)	25 (3.5%)	28 (2.5%)

Cause of the death was obtained from pig caretakers’ records in addition to veterinary post-mortem examinations when possible. N: initial number of pigs at weaning. In brackets, percentages are based on the total number of pigs in the herd at weaning

**Fig. 2:**
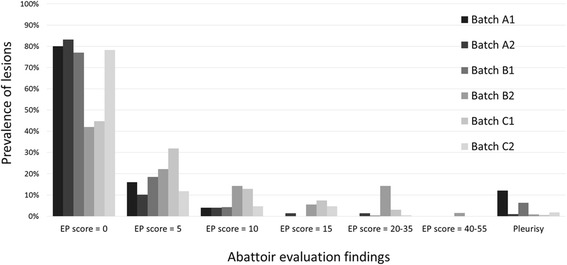
Respiratory system lesions in abattoir evaluations. Frequency distribution of Enzootic Pneumonia (EP)-like lesion scores based on the Goodwin scale [36], and prevalence of pleurisy lesions were assessed for a subset (*n* = 126 – 250 pigs) of each study batch

Lung lesions were evaluated at slaughter in 126 to 250 pigs per batch (Table 2). Batch level average EP-like lesion scores ranged from 1.2 (A1) to 7.8 (B2) (Table 2); frequency distribution of EP-like lesion scores is shown in Fig. 2. The prevalence of pleurisy lesions detectable at slaughter ranged from 1% (C1, A2, B2) to 12% (A1) (Table 2, Fig. 2).

### OF sample collection

Samples were successfully collected on a total of 310/320 scheduled pen sample time points. The principal reasons for failure to collect a sample were low levels of pig interaction or damage to the sampling material caused by destructive interaction. Lack of interaction with the ropes was a major problem for recently weaned piglets and young pigs in cold weather.

### Laboratory analysis of OF and serum samples

#### PRRSV

**Fig. 3:**
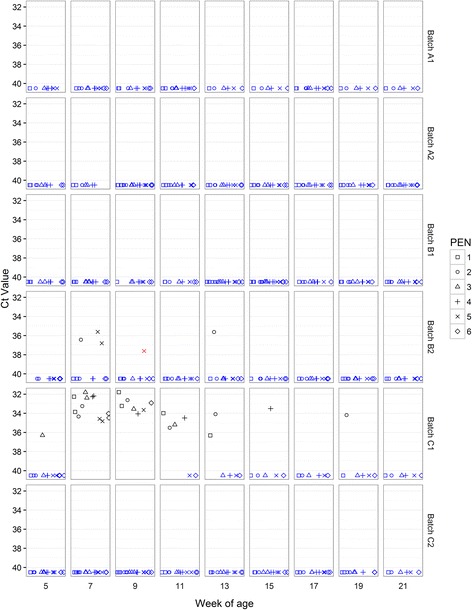
PRRSV Ct values in OF samples. Real-time PCR Ct values for PRRSV in OF samples collected from pens at between 5 and 21 weeks of age. Each sample from each rope is represented regarding batch, time point and pen from which it was collected. Samples with positive detection are represented in *BLACK* (Ct values <37); inconclusive results in *RED* (Ct values ≥37 and <40); Negative results (no detection in CT ≥ 40) in *BLUE*

PRRSV nucleic acid was detected only in C1 (7/9 time points) and B2 (3/9 time points) (Fig. 3) with positive results obtained from weaning age onwards in both batches. Maximum pen-level prevalence was 6/6 for C1 but only 2/6 for B2. There was an apparent decline in pen level prevalence at later time-points. Detection patterns were irregular over time in that a pen testing positive on multiple occasions might also report interspersed negative results. All Ct values were higher than 31 indicating low levels of detectable viral RNA in the samples. Serum samples collected at 15 weeks of age were negative for PRRSV PCR for every batch, and positive for PRRSV antibodies in 12/12 pigs tested in each of C1 and B2 but all other batches gave negative results (Table 4).

**Table 4:** Results of PRRSV antibody ELISA analysis and PCV2 qPCR in serum at 15^th^ week

Farm/Batch	PRRSV antibody ELISA (*n* = 12 pigs per batch)	PCV2 qPCR (*n* = 12 pigs per batch)
A1	Negative (0/12)	Negative (0/12)
A2	Negative (0/12)	Negative (0/12)
B1	Negative (0/12)	Negative (0/12)
B2	Positive (12/12)	Inconclusive (4/12; 10^2.5^ to 10^3.5^ PCV2 genome copies/mL, values under the limit of detection)
C1	Positive (12/12)	Negative (0/12)
C2	Negative (0/12)	Negative (0/12)

#### PCV2

**Fig. 4:**
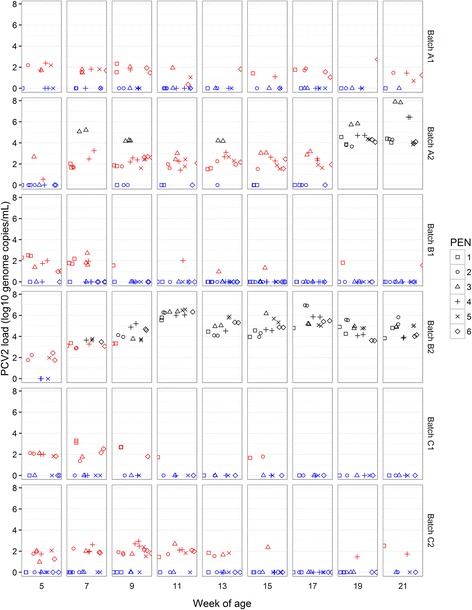
PCV2 viral load in OF samples. PCV2 viral load (log10 genome copies/mL) for OF samples collected from pens at between 5 and 21 weeks of age. Each sample from each rope is represented regarding batch, time point and pen from which it was collected. In *BLACK* viral load values over the limit of detection (1 × 10^3.48^ genome copies per mL). In *BLUE*, negative samples where PCV2 DNA was not detected. In *RED*, inconclusive results with viral load values under the limit of detection. Note that viral load values under the limit of quantification (1 × 10^4^ genome copies per mL) could be not accurately quantified

PCV2 DNA amplification was apparently detected by qPCR in OF at most sampling occasions across the study, ranging from 5/9 occasions for C1, through to 9/9 sampling occasions for A1, A2 and B2 (Fig. 4).

Three different patterns of detection were found. First, batches A1, B1, C1 and C2 recorded viral loads under the limit of detection of the method. There was a trend among these four batches for reduced apparent detection prevalence notably after the 13^th^ week of age (Fig. 4).

Batches A2 and B2 each showed different patterns of PCV2 detection in OF. Both these batches recorded higher virus loads over the limit of detection on 5/9 and 8/9 sampling occasions respectively, with the positive pen prevalence being lowest at the 5^th^ week of age. However, A2 differed from B2 in that only one pen in A2 was responsible for yielding OF samples over the limit of detection until the 19^th^ week after which time all pens exceeded this value at the 19^th^ and 21^st^ week, with a peak value of 1 × 10^7.9^ genome copies/mL in the 21^st^ week. Conversely, for B2, OF samples from all pens exceeded the limit of detection at all sampling points from the 9^th^ week onwards, peaking at 1 × 10^7^ genome copies/mL in the 17^th^ week of age and this batch also recorded the highest number of casualties (see Fig. 1 and Table 3).

Serum samples collected in the 15^th^ week of age and every sample had PCV2 viral load values under limit of detection for serum (Table 4).

Post-mortem examinations reported combinations of gross lesions compatible with PCV2 including wasting, emaciation, pallor, rough coat, ascites, jaundice, discoloured liver, interstitial pneumonia, lymph node enlargement and interstitial nephritis [34, 39] in pigs from B2 while examinations on casualties from A1, B1, C1, A2 and C2 did not present suspicious combinations of lesions. Histopathological analysis and immunohistochemistry (IHC) were done on any casualty pigs with individual signs that might be suspicious of PCVD. Clinical PCVD diagnosis was confirmed by histopathology and IHC in batch B2 (4/14 pigs tested; all positive cases were from the 9–15 weeks-old grower stage). In addition 1/2 pigs tested in A2 showed patchy IHC staining for PCV2 antigen. No positive cases were reported in A1, B1 and C1 where IHC testing was done in 2, 1 and 3 pigs respectively. No pigs in C2 were tested for IHC.

#### SIV

**Fig. 5:**
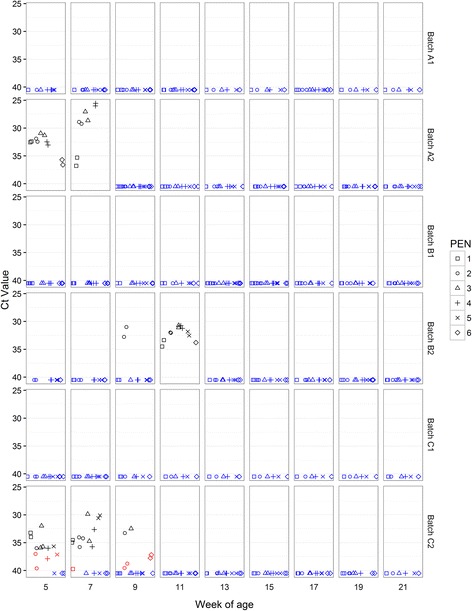
Swine influenza virus Ct values in OF samples. Real-time PCR Ct values for SIV in OF samples collected from pens at between 5 and 21 weeks of age. Each sample from each rope is represented regarding batch, time point and pen from which it was collected. Samples with positive detection are represented in *BLACK* (Ct values <37); weak positive results in *RED* (Ct values ≥37 and <40); Negative results (no detection in CT ≥ 40) in *BLUE*

SIV nucleic acid was detected in OF at two consecutive sample points in A2 (5^th^ and 7^th^ week of age) and B2 (9^th^ and 11^th^ week of age) and 3 consecutive sample points in C2 (5^th^ to 9^th^ week of age) (Fig. 5). The prevalence of positive pens ranged between one and five out of six. Of the three positive batches, clinical signs including respiratory problems, cough, sneezes, fever, and prostration, and lesions including interstitial pneumonia, multifocal catarrhal pneumonia; all compatible with influenza were observed only in B2. Sub-typing of all positive samples resulted in classification as H1N2.

#### *M. hyo*

**Fig. 6:**
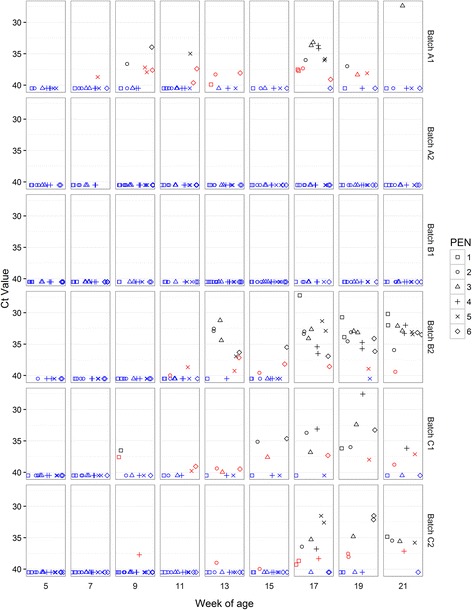
*Mycoplasma hyopneumoniae* Ct values in OF samples. Real-time PCR Ct values for *M. hyo* in OF samples collected from pens at between 5 and 21 weeks of age. Each sample from each rope is represented regarding batch, time point and pen from which it was collected. Samples with positive detection are represented in *BLACK* (Ct values <37); weak positive results in *RED* (Ct values ≥37 and <40); Negative results (no detection in CT ≥ 40) in *BLUE*

*M. hyo* nucleic acid was detected in OF in A1, B2, C1 and C2 (Fig. 6). All batches yielded consistently negative results just after weaning (5^th^ week of age). Detection patterns were irregular over time in that a pen testing positive on multiple occasions might also report interspersed negative results. Positive pen prevalence increased from the 17^th^ week of age onwards with, notably, all six pens in B2 giving a positive result at all three of the final sample points before slaughter (17^th^, 19^th^ and 21^st^ week of age). Ct values were variable with a minimum Ct value (between 27 and 28) recorded in batches B2 (at the 17^th^ week of age), C1 (at the 19^th^ week of age), and in A1 (at the 21^st^ week of age). These batches with the greatest number of pens positive for *M. hyo* across the 19^th^ and 21^st^ week of age sample points to presented the most severe EP-like lesion scores at slaughter (Table 2).

#### Correlation between multiple OF samples collected from the same pen

Two or more OF samples were collected in 219 pens (generating 484 OF samples) while single OF samples were obtained from the remaining 91 pens resulting in a total of 310 tested pens and 575 OF samples analysed.

Ropes collected at the same time in the same pen showed similar Ct values on most occasions; agreement (same results) for multiple OF samples collected from different ropes in a pen, with at least one positive rope-sample, was 67% for PRRSV (8/12 positive pens), 96% for PCV2, (51/53), 78% for SIV (21/27), and 53% for *M. hyo* (18/34).

**Fig. 7:**
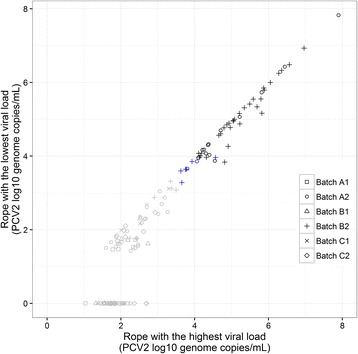
PCV2 viral load for OF samples collected in the same pen. PCV2 viral load logarithmic values for pairs of OF samples collected from the same pens in the same time point. For each pen the rope with the highest and the lowest viral load were included. In *BLACK*, viral load over the limit of quantification for both ropes; as it has been not defined for OF, the authors considered the reference value for serum (1 × 10^4^ genome copies/mL). In *BLUE*, PCV2 positive samples with at least one rope over the limit of detection (1 × 10^3.48^ genome copies/mL) but under limit of quantification (1 × 10^4^ genome copies/mL). In *GREY*, PCV2 viral load values under the limit of detection for at least one rope; note that viral load values under the limit of quantification could be not accurately quantified. There was a strong correlation (*R* ^2^ = 0.97, p < 0.01) between viral load for pairs of ropes with PCV2 viral load over the limit of detection

In those pens where multiple samples tested positive or weak positive (in the case of SIV or M. hyo), correlations between different samples Ct values were significant (*P* < 0.01) and strong (*R* ^2^ ≥ 0.60) for every pathogen tested PCV2 (*R* ^2^ = 0.97) (Fig. 7), PRRSV (*R* ^2^ = 0.85), SIV (*R* ^2^ = 0.79) and *M. hyo* (*R* ^2^ = 0.60).

#### Agreement among pens in a barn

A total of 54 “barns” -six barns in nine time points- were tested; 10 (19%) were positive for PRRSV, 13 (24%) were positive for PCV2, seven (13%) were positive for SIV and 18 (33%) were positive for *M. hyo.*

Agreement among the tested pens in a barn was low; PRRSV was detected in just a single pen in 50% of the cases (five out of 10 positive barns), 23% for PCV2 (three out of 13 positive barns), 14% (one out of seven positive barns) for SIV and 28% (five out of 18 positive barns) for *M. hyo*. Three or more pens out of six in a barn were positive at the same sample time point in 30% (three out of 10) of the positive barns for PRRSV, 77% (10 out of 13 positive barns) for PCV2, 71% (five out of seven positive barns) for SIV and 50% (nine out of 18 positive barns) for *M. hyo*.

#### Consistency of pen results across time, and spatial patterns or sex effects

Detection patterns for the same pen across time were irregular especially in the case of *M. hyo*; a pen that was positive in a given time could be negative in the next sampling and positive again later on (Fig. 6). However in the case of PCV2, detection patterns were more stable and those pens with higher viral load tended to present similar results at subsequent sample points (Fig. 4).

No differences in detection patterns were seen between pens in terms of spatial distribution within the building or in terms of sex distribution.

## Discussion

OF sample collection was found to be straightforward under most on-farm conditions. Sampling was difficult in younger pigs in the three days after weaning, especially when pigs were not previously exposed to ropes; this problem was more pronounced when environment conditions were cold and piglets tended to huddle. In older ages, the main cause of sample collection failure was destruction of the sampling ropes by aggressive chewing or a lack of interaction. Therefore planning of OF-based sampling strategies should take account of timing and environmental conditions in order to allow sampling to be done when pigs are most likely to interact with the collection ropes.

Five out of six batches presented respiratory problems. Severity of clinical disease was not directly related to the initial classifications; multiple post-weaning management factors at the growing farm could have had an important impact on pigs’ health. It was notable that clinical signs of diseases suspected to be caused by PCV2, *M. hyo* and/or PRRSV were observed despite vaccination. All vaccinations were made on the 4^th^ or 5^th^ week according to the practices at each farm; however, results of OF analyses for PRRSV (in C1) and PCV2 (in A2 and B2) suggest that viral circulation started soon after weaning indicating that vaccination timing was possibly non optimal and a contributor to clinical disease.

The observed patterns of detection of PRRSV, SIV and *M. hyo* highlighted opportunities and limitations for the use of OF testing as part of a diagnostic approach for PRDC. In terms of PRRSV detection, previous studies already reported the successful use of OF to detect PRRSV in young pigs [40] but our findings emphasised the benefit of sampling from multiple pens on a repeated basis in order to overcome recognised limitations in sensitivity of detecting PRRSV in pooled OF samples [41]. On the other hand, our findings showed the usefulness of pooled OF samples for detection of both clinical and non-clinical SIV infection with a window of two to four weeks for detection, confirming the previously reported prolonged shedding of SIV in OF [42]. Although this study showed the usefulness of OF testing for confirmation of *M. hyo* in clinical PRDC, including in two batches (A1, B2) derived from sources believed to be *M. hyo* negative based on clinical history, it emphasised the limited sensitivity of this testing method. Results presented negative detection by PCR in pig groups that previously, and subsequently had positive detection which suggests this testing method has a poor sensitivity. Therefore the absence of detection of *M. hyo* nucleic acid in pooled OF should not be interpreted as absence of infection from the population. In addition, we found a tendency for those batches with the greatest number of pens positive for *M. hyo* across the 19^th^ and 21^st^ week sample points to present the most severe EP-like lesion scores at slaughter which suggests that higher prevalence and lower Ct values could be related to respiratory problems with *M. hyo* active involvement. The results in this study emphasized the potential value of OF as a sampling platform to study pen-level prevalence and PCV2 viral load in pooled pen-level samples, as previously described [14, 26]. Even though no relationships between PCV2 detection in OF and prevalence or severity of respiratory disease were apparent, the data indicated the possible use of pooled OF samples to support the diagnosis of PCVD at population level (see Fig. 4 and Table 2). The current study was limited by the small number of participating farms so observed relationships between timing and load of PCV2 detection in pen-level OF samples and results of confirmatory diagnostic tests for PCVD in those farms must be interpreted with caution. In addition, interpretations of quantitative data on PCV2 load in pooled OF samples must be made with caution due to uncertainties including the number of animals contributing to the pool and their individual level of shedding. Nevertheless, previous studies of individual pigs found relationships between the quantities of PCV2 in various samples with generally increased viral load detectable for pigs with clinical PCVD, including in OF [43–46]. Most of these studies associate serum viral load and PCVD; correlations between PCV2 viral loads in serum and individual oral fluids were reported as strong (*R* ^2^ = 0.6) [26], and oral fluids were described as more sensitive than serum to detect PCV2 when blood samples only represent fraction of the group [47].

Differences in viral loads and patterns of detection at pen level were observed between farms with and without PCVD problems. Farms without detectable clinical or subclinical PCVD in this study presented consistently low viral load in pooled OF samples, generally below the limit of detection, and the number of samples without any PCV2 detection increased with age (Fig. 4). In contrast, higher levels of PCV2 were detected in OF, already exceeding the limit of detection consistently across sampled pens by the 5^th^ week of age, where clinical PCVD was confirmed from the 9^th^ week onwards by clinical signs, gross pathology and IHC for PCV2 antigen (Batch B2). In contrast, a different detection profile was found in one batch (A2) where some evidence for subclinical infection by PCVD was found by IHC staining of viral antigen in lymph node but evidence of clinical PCVD was not found. Here, there was much less consistency in detected viral load between pens sampled at the same time point, with consistently high PCV2 viral load being detected only from the 19^th^ week onward. These findings emphasise the potential value of further more extensive studies using qPCR on pen-level OF samples to explore further any associations between shedding load of PCV2, clinical and subclinical PCVD, and productivity parameters.

Our findings supported sampling designs that target as many pens as possible, rather than focusing resources into collecting multiple rope based OF samples from a smaller number of pens. Close correlation of Ct values between two positive OF samples from the same pen supported this, as did the finding that the proportion of detectably positive pens on a given sampling occasion could be as low as one out six pens. Further studies could support objective determination of the sensitivity of pen level OF testing for respiratory pathogens, compared to other individual sample platforms such as serum or nasal swabs. Direct comparisons are complicated by uncertainties including the relative contribution of individual animals to a pooled sampled, differences in individual shedding load between individuals, as well as differences in shedding of the target pathogen by different routes.

## Conclusions

This study provided practical information on the design of OF sampling strategies to support on-farm investigations of respiratory disease in pigs involving PRRSV, SIV, *M. hyo* and PCV2. Importantly, sampling design needs to account for limitations in sensitivity of the test and for differences in herd level infection dynamics. Finally, we found preliminary evidence that measurement of PCV2 load in pooled OF might serve as a tool for prediction of clinical or subclinical PCVD at farm level.

## Abbreviations

BALF: Bronchoalveolar lavage fluid
BPHS: British Pig Executive Pig Health Scheme
Ct: Cycle threshold
DNA: Deoxyribonucleic acid
EP: Enzootic pneumonia
IHC: Immunohistochemistry
*M. hyo*: *Mycoplasma hyopneumoniae*
OF: Oral fluid
PCR: Polymerase chain reaction
PCV2: Porcine circovirus type 2
PCVD: Porcine circovirus diseases
PRDC: Porcine respiratory disease complex
PRRSV: Porcine reproductive and respiratory syndrome virus
qPCR: Quantitative polymerase chain reaction
RNA: Ribonucleic acid
SIV: Swine influenza virus
